# Perspective: Daylight Saving Time—An Advocacy for a Balanced View and against Fanning Fear

**DOI:** 10.3390/clockssleep2010003

**Published:** 2020-01-19

**Authors:** Christine Blume, Manuel Schabus

**Affiliations:** 1 Centre for Chronobiology, Psychiatric Hospital of the University of Basel, 4002 Basel, Switzerland; 2 Transfaculty Research Platform Molecular and Cognitive Neurosciences, University of Basel, 4055 Basel, Switzerland; 3 Centre for Cognitive Neuroscience, University of Salzburg, 5020 Salzburg, Austria; 4 Laboratory for Sleep, Cognition, and Consciousness Research, University of Salzburg, 5020 Salzburg, Austria

**Keywords:** daylight saving time, standard time, social jetlag, clock change

## Abstract

As experts, scientists must inform the public and political actors about relevant topics by providing a well-balanced analysis and overview of *existing* as well as *missing* scientific evidence. Particularly in cases where evidence is not solid, they must remain objective and not fan fear. Maintaining good scientific practice can be challenging, especially when a debate is emotionally charged and simple answers for complex issues are demanded. Recently, this was the case with the debate about (perennial) standard time vs. daylight saving time. In this publication, we address the common misconceptions and pitfalls for good scientific practice that accompany this discussion and deduce suggestions for future directions, which may help resolve them. Beyond this, we argue that it is not wise to simply “explain away” the public opinion or preference and we therefore recommend strategies that could support a discourse aiming at getting the public “on board”. Finally, we suggest that, in societies where the light environment is becoming increasingly complex, it may be time to reconsider the prevailing current relationships between solar and social clocks.

## 1. Introduction

In autumn 2018, an announcement from the European Commission President that the bi-annual clock change in Europe would be abolished triggered a debate about whether Europe should choose perennial standard time (ST) or daylight-saving time (DST). In this context, scientists also voiced their opinions on what would be the better choice. Some researchers warned against enormous problems this would cause for public health and well-being, a perspective that was also well-perceived by the media [1,2]. However, other researchers, including us, felt that this conclusion resulted from a rather one-sided and selective interpretation of studies that almost exclusively present *indirect* evidence, that is, they did *not* compare ST and DST, wherefore the results warrant interpretation. Therefore, we and others argued that the available scientific evidence should not translate into such a fatal vision of a future under perennial DST [3]. We would like to emphasise that we believe diverging interpretations and opinions are a natural and necessary driving force for scientific advance and that the freedom of opinion is certainly a precious commodity in science. However, we also believe that good scientific practice needs to consider all available evidence, acknowledging, in particular, the missing evidence that is required for a well-balanced assessment. In line with this, we think that our task as scientists is to provide the public with a balanced view on topics that cannot easily be accessed by lay people. This must include adequate communication of the uncertainties scientific findings naturally bear. Finally, our task as scientists is *not* to fan fear, particularly when evidence is not solid or ambiguous. Providing such a well-balanced perspective can be challenging, especially when a discussion is emotionally charged, and scientists are asked to provide simple answers to complex problems—as was the case with the DST/ST discussion. Unfortunately, several misconceptions and shortcomings in the arguments commonly employed in the scientific discussion of this topic have become evident. Here, following clarification of the scientific consensus position regarding perennial DST/ST, we would like to address the most important misconceptions and recommend steps for future directions that may help to resolve them. Beyond this, we will argue that it is necessary to bring political actors, scientists, and lay people together for the outcome of the debate to be most beneficial to society. Eventually, we suggest that it may be time to reconsider the prevailing relationship between solar and social clocks.

## 2. Current Consensus Position

The current consensus position is that the risk of negative effects on, for example, public health and safety would be higher under perennial DST than under perennial ST ([4,5] 2019; Dijk, Vandewalle, Winnebeck, Wright Jr, and Blume, 2018: consensus conclusions from a panel discussion at the meeting of the 2018 European Sleep Research Society meeting). Importantly though, researchers also recognise that “at this time […] there is no direct evidence to reliably determine the magnitude of the health and safety effects […]” [5].

## 3. Misconceptions and Pitfalls

### 3.1. Do We Need More Research?

Calls for a balanced perspective in the DST/ST debate, which must also highlight missing evidence, have repeatedly been countered with the objection “you just ask for more research”, which has sometimes even been called a “dangerous” position (personal communication of the authors). First, like a perpetuum mobile, research will naturally always generate new questions and result in the need for more research. Critically though, this objection seems to be used to argue that, in cases where evidence from existing research is not solid, adopting a one-sided perspective was justified. While we agree that political actors need scientists’ advice in the DST/ST debate *now*, this does *not* justify a one-sided perspective. On the contrary, it underlines the importance of adequately communicating what scientists do (not yet) know. Especially in an emotionally charged debate, where scientists’ recommendations might clash with people’s preferences and perhaps even with the results of a referendum, we otherwise risk squandering scientists’ credibility in the long run.

### 3.2. What Can We Say about the “Level of the Individual”?

Most scientific publications do not, due to their design and statistical analysis, allow for interpretations on the “level of the individual”. This becomes especially relevant when communicating the risks of, for example, a disease and its association with certain (risk) factors. In the DST/ST discussion one question, for example, is whether the negative effects of the transition to DST in spring on, for example, the risk of myocardial infarction, are balanced out by the positive effects that have been reported for the switch to ST in autumn [6] (note that a recent meta-analysis by [7] suggests negative effects following the transition to, but no effects out of, DST). Here, in a recent publication Roenneberg et al. [8] state that “the (negative, authors’ note [A/N]) spring and the (positive, A/N) autumn effect do not balance each other out on the *individual* level”. While this may theoretically be true, it is misleading in two ways. First, it suggests that myocardial infarction in an individual can be prevented by abolishing clock change. In the same vein though, one could also argue that the change from DST to ST in autumn saves lives. Importantly, both claims would, however, require that myocardial infarction or its prevention can, beyond doubt, be attributed to this event. As this will hardly ever be possible, the statement is neither verifiable nor falsifiable. Second, any conclusion about the level of the individual is not allowed from group-level analyses [9,10]. An intuitive example from Hamaker [11], illustrating this problem, is the relationship between typing speed and typos. On the group level, the correlation between speed and typos is negative, as more experienced typists are faster and make less mistakes. At the individual level though, the correlation is positive: the faster a given individual types, the more mistakes she or he will make compared to the performance at decreased speed. Besides this, it should be noted that even odds ratios, which are frequently reported, do not provide a simple quantification of the risk of an individual, wherefore they are often misinterpreted [12]. Additionally, they frequently magnify an existing effect [12]. To inform about the risks threatening individuals, they need to be converted to relative risks, considering the baseline risk of the outcome [13].

### 3.3. Statistical Effect Sizes vs. Practical Meaningfulness of Effects

Scientific studies are usually designed to find an effect of factor A (e.g., a newly identified risk factor) on dependent variable B (e.g., outcome). In order to isolate such an effect, the experimental design controls for confounding factors, for example by keeping them constant. Such studies allow for conclusions regarding whether factor A has an effect on the outcome B, and statistical effect sizes allow for a quantification of an effect in this very setting independent of statistical significance and sample size. While reporting effect sizes is nowadays, in many fields, the norm rather than the exception, their interpretation often remains challenging. This is because, as we will explain below, statistical effect sizes do not necessarily *directly* translate to the practical relevance of an effect (e.g., whether risk factor A is practically meaningful). Moreover, they do not, unless the study design is otherwise identical, inform about the relative meaningfulness of factor A in comparison with factor C (e.g., a well-known risk factor). Such a comparison could, however, also provide key information about a factor’s practical meaningfulness.

In the DST vs. ST debate, the only empirical study directly investigating the effects of perennial DST and ST found, for example, that DST was associated with an increase in “social jetlag” (SJL) [14], which is defined as the difference between the mid-sleep point on workdays and free days. Repeatedly, SJL has been found to be a risk factor for higher body mass index (BMI) or even obesity, depressive symptoms, and for behaviour that is hazardous to health, such as smoking and poor dietary habits (for a review see [15]). The effects found by Borisenkov et al. (2017) [14] correspond to a statistically small to medium effect size (*r* = 0.2). Roenneberg, Winnebeck, et al. [8] however recently claimed that this effect was “biologically large”. While that may be true, such a claim is unjustified so long as we lack information about the practical meaningfulness of differences in SJL on an interval scale, that is, so long as we lack knowledge about how a certain change in of SJL (e.g., of 20, 30, or 40 min) relates to a change on an outcome variable (e.g., symptoms of seasonal affective disorder or body mass index). Such a judgment would require studies that go beyond just correlations and investigate the causal relationships between SJL and outcome variables. Ideally, results of such studies should even be reported in terms of relative risks rather than, or at least in addition to, effect sizes [16]. Here, we would also like to suggest an alternative to statistical effect sizes that could help appreciate the practical meaningfulness of certain factors, such as time zone position (which can serve as a proxy for ST/DST) in epidemiological studies. More precisely, we recommend reporting the “disability/disease-adjusted life years” (DALY) [17], a measure of the overall disease burden due to ill-health, disability, or early death, where one DALY corresponds to one year of “healthy” life lost. This metric could already be calculated from epidemiological studies, such as the “Global Burden of Disease Study”, if information on time zone position is available. Then, the number of years lost due to living at the western border of a time zone (“DST-like” position) could be compared to the years lost due to living at the eastern border or in the centre (“ST-like” positions). Importantly, for an adequate appraisal of its relative and practical meaningfulness, the numbers eventually would have to be compared to DALYs due to other factors, such as occupational stress, lack of physical activity, cancer, smoking, or depression (for an example incl. methodological details see [18]).

### 3.4. Importance of the Solar Clock and Artificial Light

**Figure 1:**
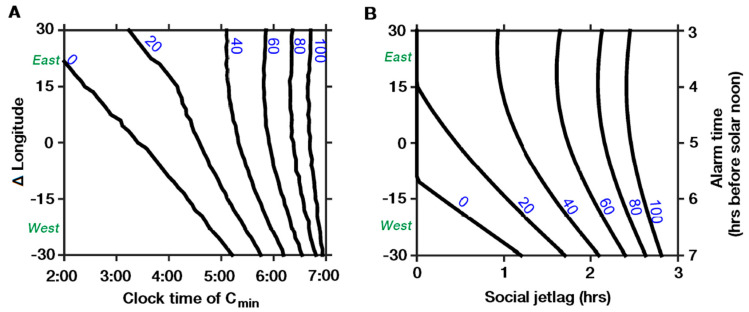
Modelled changes in circadian timing and social jetlag across a time zone for different levels of evening light (range 0–100 lux, cf. numbers on the lines). (**A**) Core body temperature minimum (C_min_) indicating the minimum of the circadian wake propensity. C_min_ occurs later in western parts of a time zone at low levels of evening light, as indicated by the smaller slope of the lines. With increasing levels of evening light though, the effect of the time zone position disappears, as indicated by the steepening slope. (**B**) Social jetlag is larger in the western parts of a time zone (cf. smaller slope) but decreases with increasing levels of evening light (cf. increasing slope). Note that a 15° shift to the east (west) within a time zone corresponds to an advance (delay) in solar noon by one hour relative to clock time. Abbreviations: C_min_ = core body temperature minimum; hrs = hours. Adapted from Skeldon et al. [22].

On a less methodological note, there is universal agreement that light is the strongest zeitgeber for the biological clock [19]. Earlier studies concluded that the solar clock determines bedtimes, and thus sleep duration and SJL, when get-up times are constant on workdays. In recent years however, the effects of artificial light, in addition to natural sunlight, have gained increasing interest, particularly with the advent of light-emitting diodes (LEDs; see e.g., Blume, Garbazza, and Spitschan [20]) whose light spectrum is “circadian active”. Moreover, natural daylight plays a decreasing role in modern societies, with some authors even arguing that many people live in “biological darkness” [21]. In the DST/ST debate, this is a crunch point as it relates to the question of how much the body clock depends on the apex of the sun’s motion coinciding with 12 pm/noon in a world with an increasingly complex light environment. In their recent review, Roenneberg, Winnebeck, et al. [8] state that “the combination of night-time light exposure and DST is far worse than night-time night (should be “light”, A/N) exposure alone.” While, from the current body of literature, it is not possible to support such a priority claim, recent research suggests that the availability of artificial light in the evening may rather mitigate than potentiate the effects of later solar noon (as under DST). More precisely, modelling data suggest that the effect of a later solar noon under DST is modulated by artificial light consumption (cf. Figure 1; adapted from [22]). Here, the higher light levels were in the evening, the less strong were the effects of a shift in the solar noon (expressed by longitude differences) on SJL and the occurrence of the body temperature nadir.

Further support comes from a recent publication by Shochat et al. [23]. Here, the authors conclude that “individual timing of sleep and activity in a modern environment with electrical lighting, largely conforms to clock time and actual light exposure (which is the combination of natural and electrical light exposure) rather than sun time indexed by the timing of SN (solar noon, A/N)”. Note that, although the findings do not imply causality and the timing of sleep also influences the timing of light exposure, any light exposure will impact the circadian pacemaker and thereby sleep timing [22,24].

## 4. Role of the “Public Preference”

Beyond a necessary scientific discussion, the DST/ST debate should, in our opinion, not just ignore the public preference and scientists should not just try to simply “explain it away”. While scientists *are* the experts and the risk for negative effects *does* seem to be higher with perennial DST, pressing for a policy simply because “we know better”, especially when the public has been asked for its opinion beforehand, has usually not proven wise. Therefore, political actors and scientists should take great care to create understanding for a, perhaps unpopular, decision.

In an EU survey, in which <1% of the population of the EU (70% German) participated, 84% of the respondents voted to abolish clock change. If it was abolished, 56% would keep perennial DST whereas 36% would favour perennial ST, with only 8% indicating no preference (for survey results see [25]). In addition to this, relative to the size of the European population small and biased sample, it seems important to note that those who were not interested in the topic, wanted to keep the status quo, or simply did not care about the outcome, did not participate [26]. While it is debatable whether the results do reflect the “public preference”, most respondents in favour of perennial ST (43%) mentioned general health considerations and the main argument (43%). The main argument (42%) for perennial DST was evening leisure time activities, which are facilitated by the prolonged availability of daylight. In short, irrespective of scientific considerations, some people feel that DST, whether perennial or during the summer months, increases their quality of life. And they might be right in prioritising social and leisure time activities. This is because social relationships and activities have been shown to be a protective factor against many health-related problems, including cognitive decline in dementia [27,28]. Besides this, although two studies that took the geographical position of a region into account suggested that health risks, such as cancer incidence and mortality, could increase in more western parts of a time zone (with DST equalling a shift to a more western position) [29,30], a study in Europe did not provide support for this notion [31]. Thus, at the moment, this debate seems unsettled. Irrespective of this, social relationships, which people feel benefit from DST, have also been associated with increased cancer survival rates [32,33].

Moreover, the simple claim that (i) the preference for DST in the EU survey only resulted from a preference for the word “summer” (note that there is no neutral alternative for the German term “Sommerzeit”, i.e., “summer time”) and (ii) that, if the EU survey had been conducted during winter, the majority would have preferred ST [1,8], does not seem to hold true. A survey in Austria that, off its main focus, included questions on the attitude towards clock change between September 2018 and May 2019, i.e., during a sampling interval excluding the summer months, found an even higher preference for perennial DST compared to the EU survey. From the 58% that would abolish clock change, 58% would prefer perennial DST and 40% would vote for perennial ST [34]. Note that a possible reason for this result could include Austria’s geographical location in central Europe. However, this highlights the fact that survey results cannot simply be “explained away” and ignored.

## 5. Concluding Remarks

In conclusion, although the magnitude of the potential effects of perennial DST cannot be reliably determined from the available data, the current state of research suggests that the *risk of negative effects* associated with perennial DST is higher than with perennial ST [5]. Especially in cases where the *magnitude* of potential detrimental health effects is yet to be determined from solid empirical data, scientists need to be particularly careful about *what* and *how* they communicate their recommendations to the public. In this context, it also seems important to prevent the impression that they put themselves before people and ignore their concerns and preferences. This seems particularly relevant in times when scientists’ authority and credibility is challenged publicly even by leading political actors. We therefore encourage a discourse, which aims to get the public on board, that could include think tanks led by independent institutions, such as the National Academy of Sciences Leopoldina (in Germany). Eventually, in societies where artificial “circadian-active” light is so readily accessible and we spend most of the day indoors, we should welcome and encourage a broader discussion about the adequacy of the prevailing timing of social schedules relative to natural light–dark cycles (the “solar clock”). This seems especially timely as the EU survey indicated that evening leisure time activities are an important reason to favour perennial DST.
